# Mandible giant-cell reparative granuloma

**DOI:** 10.1016/S1808-8694(15)30502-4

**Published:** 2015-10-19

**Authors:** Michelle Manzini, Christian Deon, Liliam Dalla Corte, Luciana Boff de Abreu, José Carlos Bertotto

**Affiliations:** 1 4th Year Medical Student - Universidade de Caxias do Sul; 2 4th Year Medical Student - Universidade de Caxias do Sul; 3 4th Year Medical Student - Universidade de Caxias do Sul; 4 4th Year Medical Student - Universidade de Caxias do Sul; 5 Full Professor of Otorhinolaryngology and head of the Ophthalmo-ENT Programs - Medical School - Universidade de Caxias do Sul. Universidade de Caxias do Sul

**Keywords:** mandible, neoplasm, tumor

## INTRODUCTION

Giant cell granuloma of the respiratory tract or Central Giant Cell Granuloma (CGCG) is considered an intraosseous, non-neoplasm tumor, responsible for less than 7% of all the mandible expansive lesions, its most common site of involvement.^1^, ^2^, ^3^

It affects more often the young people – 75% of the cases happen to patients below 30 years of age.^4^ There is a greater predilection for females.^5^

This paper describes a CGCG case, with mandible involvement and revises characteristics inherent to CGCG, with emphasis in diagnosis, thus yielding a less mutilating treatment.

## CASE REPORT

A 30 year old brown woman came to our ENT outpatient ward because of a recurrence of a left mandible tumor, with a progressive, painless growth, associated with facial deformity after a surgical removal of a giant cell reparative granuloma in the previous year.

She reported a prior dental removal, 11 years before in the left mandible region. She denied past diseases.

The physical exam showed a hard and painless tumor, a bone notch in the left mandibular region associated with facial deformity.

Routine workup, serum calcium, phosphorus and alkaline phosphatase were normal.

The panoramic radiograph showed a radiolucent area in the left mandible body, a recent post-surgical area, with mild radiologic signs of bone regeneration.

**Figure 1:**
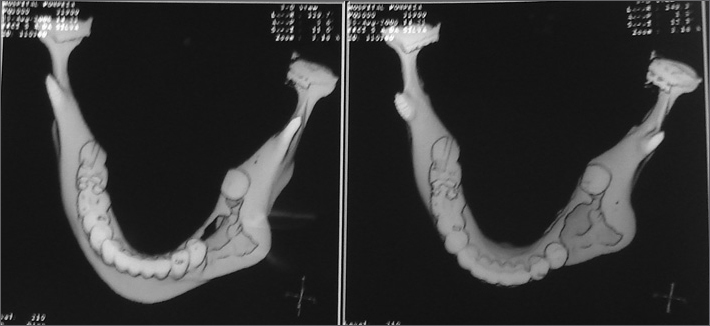
Mandible CT scan showing morphostructural alteration with bone remodeling and a large irregular lithic area in the body and alveolar processes of the left mandible.

The mandible CT scan showed a morphostructural alteration with bone remodeling and a large irregular lithic area in the mandible body and left mandible alveolar processes which could correspond to post-surgical alteration or tumor recurrence (Figure 1).

The tumor was surgically excised without complications.

The pathology exam confirmed central giant cell granuloma.

## DISCUSSION

The mechanism by which these lesions are formed is still uncertain. Local causes suggested are trauma and vascular injuries, and the systemic causes reported in the literature associate the development of CGCG with syndromes such as, Neurofibromatosis I, Noonan syndrome and hormonal disorders such as hyperparathyroidism and pregnancy.^4^

The clinical manifestation can evolve in a few weeks or take years, it is usually associated with tumor expansion and its respective compressive effects on adjacent structures, with a possibility for local discomfort; however, overt pain is less frequent. Clinically speaking, the differential diagnosis of CGCG goes from a cyst all the way to a malignant lesion, and one must investigate the level of aggressiveness, how fast it develops, inflammatory characteristics, pain and dental mobility.^4^

CGCG radiographic characteristics are not pathognomonic, they are radiolucent uni or multilocular images, well outlined and with peeled off margins.^5^One important aspect used to define lesion location and extension and to plan CGCG treatment is the ordering of a CT scan.^1^,^4^

Radiographically speaking, differential diagnoses are cysts, periapical granulomas, ameloblastomas, keratocysts, myxomas and sarcomas.

Histology shows giant cells distributed throughout the loose connective tissue^4^. Based on the histopathology exam, other diseases that may present characteristics which are undistinguishable from GCGCs, such as giant cell tumors, brown hyperparathyroidism tumor, aneurismal bone cyst, cherubism and fibrous dysplasia.^4^

It is important to study levels of calcium, phosphorus, alkaline phosphatase and parathormone to rule out hyperparathyroidism.^4^

Prognostics are favorable and there are no reports of metastasis, which confirms the benign characteristic of the lesion.^4^The aggressive types of tumor, have a greater rate of recurrence. The choice of treatment will depend on the patient's age, the clinical characteristic of the lesion and its aggressiveness.

The treatment of choice for CGCG is surgical.^1^ Post-operative recurrence is 4 to 12%, and it is usually associated with incomplete tumor resection.^2^

Curettage is the most used treatment, because of its ease of execution and low grade of recurrence. Usually, the same recurrences respond well to a new curettage, unless the lesion is aggressive. For more aggressive lesions, or those that perforate the cortical bone and involve soft tissue, en-block resection may be indicated.^5^

Radiotherapy is contraindicated because of the potential for sarcomatous transformation that has been reported.^5^

For recurrent or extensive lesions, alternative treatments have been indicated, aiming at achieving lesion regression or termination, avoiding more invasive and mutilating surgical procedures.^4^

## FINAL REMARKS

CGCGs are a reason for much research and discussion, since there are many theories to explain its etiologic and clinical manifestations. It is a benign lesion, therefore proper diagnosis avoids mutilating radical treatments. Curattage is still used with high success rates. For extensive or recurrent lesions, treatment with intralesional injections of steroids can be useful.
